# Effects of Curcumin Supplementation on Exercise Recovery, Oxidative Stress, Inflammation, Muscle Damage, and Performance in Exercise and Sport Contexts: A Systematic Review

**DOI:** 10.3390/nu18121992

**Published:** 2026-06-19

**Authors:** Jesús Lloret-Gil, Desirée Victoria-Montesinos, Francisco Javier Martínez-Noguera

**Affiliations:** 1 Research Center for High Performance Sport, Catholic University of Murcia (UCAM), Guadalupe, 30107 Murcia, Spain; fjmartinez3@ucam.edu; 2 Faculty of Pharmacy and Nutrition, UCAM Universidad Católica de Murcia, 30107 Murcia, Spain

**Keywords:** curcumin, turmeric, oxidative stress, inflammation, muscle damage, athletic performance, recovery, randomized controlled trials

## Abstract

Background/Objectives: Curcumin has been proposed as a nutritional strategy to support exercise recovery through antioxidant and anti-inflammatory actions. However, trials differ in sport context, training status, supplementation timing, dose, formulation, and methodological control. This systematic review evaluated its effects on recovery outcomes in active individuals and athletes, with particular attention to the applicability of the evidence to real-world sport settings. Methods: PubMed, Scopus, Web of Science, SPORTDiscus, and Cochrane Library/CENTRAL were searched from 2012 to June 2026. Randomized double-blind placebo-controlled trials were eligible when they evaluated oral curcumin, curcuminoids, Curcuma-derived preparations with a specified curcumin dose, or curcumin combined only with bioavailability enhancers. Studies using artificial muscle-damage protocols, clinical populations, non-randomized designs, or combined bioactive interventions were excluded. Methodological quality was assessed using the Physiotherapy Evidence Database (PEDro) scale, supplemented by a Cochrane Risk of Bias 2 (RoB 2) assessment and a Grading of Recommendations Assessment, Development and Evaluation (GRADE) certainty-of-evidence evaluation. Owing to heterogeneity, findings were synthesized narratively by outcome domain, supplementation timing, formulation type, exercise context, and training status. Results: Fifteen trials were included. Favorable effects were reported in 6/7 studies assessing oxidative stress, 4/6 assessing muscle damage, 3/8 assessing inflammation, 3/7 assessing subjective recovery, soreness, or fatigue, and 4/8 assessing physical or athletic performance. However, effects varied substantially according to population, exercise context, biomarker selection, timing of assessment, and formulation type. The certainty of evidence was low for oxidative stress and very low for muscle damage, inflammation, subjective recovery/soreness/fatigue, and performance. Conclusions: Curcumin supplementation may support selected aspects of exercise recovery, particularly oxidative stress responses. However, these findings should be interpreted cautiously because the evidence derives mostly from small trials with heterogeneous populations, exercise protocols, supplementation regimens, formulations, biomarkers, and assessment time points. Evidence for muscle damage, inflammation, subjective recovery, fatigue, and performance remains inconsistent, and further well-controlled trials in trained and high-performance athletes are needed before practical recommendations can be established.

## 1. Introduction

Turmeric (*Curcuma longa*) is a spice widely used in Asian cuisines with antioxidant and anti-inflammatory properties, mainly attributed to its principal phenolic compound, curcumin [1]. Beyond its traditional culinary use, curcumin has gained increasing attention as a food-derived bioactive with potential applications in applied nutrition and sport recovery, particularly in the context of functional foods and nutraceutical strategies [1]. Curcumin exerts antioxidant effects, primarily through its ability to scavenge reactive oxygen species (ROS) and reactive nitrogen species, thereby protecting biomembranes from peroxidative damage [2]. Its chemical structure, characterized by phenolic hydroxyl and methoxy groups and a central methylene bridge, allows it to donate hydrogen atoms and interrupt free-radical chain reactions [3]. In addition to direct radical scavenging, curcumin may modulate endogenous antioxidant defense mechanisms by enhancing the activity of enzymes such as superoxide dismutase, catalase, and glutathione peroxidase, leading to improved systemic oxidative stress markers. Its anti-inflammatory properties appear to be mediated through inhibition of cyclooxygenase-2, lipoxygenase, inducible nitric oxide synthase, cytokine production, and transcription factors such as nuclear factor kappa B and activator protein 1 [2].

Pharmacokinetic research has consistently shown that curcumin exhibits low oral bioavailability due to limited absorption, low solubility, low dissolution rate, instability at intestinal pH, poor intestinal permeability, and extensive first-pass intestinal and hepatic metabolism [4]. After administration of crude curcumin powder at doses of 3.6 g or less, plasma concentrations remain extremely low, often below the quantification limit [4]. Co-administration with a high-fat meal appears to enhance absorption, probably by delaying gastric emptying and increasing gastrointestinal residence time, thereby facilitating dissolution [5]. To overcome poor bioavailability, multiple formulations have been developed. These include piperine-containing preparations, nanoparticle or colloidal dispersions, micellar systems, phytosomes, solid lipid particles, amorphous preparations, native turmeric matrices, and dispersion delivery systems [6].

When curcumin is metabolized by β-glucuronidase and sulfatase-related pathways, curcumin glucuronide and curcumin sulfate are formed. These conjugated metabolites may be less physiologically active because of their molecular size, rapid renal elimination, and limited membrane and blood–brain barrier permeability [7,8]. Some studies reporting curcumin bioavailability on the basis of hydrolyzed plasma samples may, therefore, overestimate bioactive curcumin exposure because hydrolysis quantifies both conjugated and free forms [9,10]. These pharmacokinetic limitations are particularly relevant in exercise and sport contexts, where any potential benefit of curcumin may depend not only on its biological activity but also on whether sufficient exposure is achieved at the time when exercise-induced oxidative and inflammatory responses occur [11].

During exercise, an inflammatory cascade is triggered through upregulation of pro-inflammatory cytokines, chemokines, and stress hormones, together with infiltration of phagocytic cells [12]. This response increases cell membrane permeability and causes leakage of intracellular enzymes such as creatine kinase (CK) and lactate dehydrogenase from muscle tissue, as well as hepatic release of acute-phase proteins such as C-reactive protein (CRP). These biomarkers are commonly used as indirect indicators of muscle damage and inflammation [12,13].

Intense physical training can also disrupt redox balance, leading to transient oxidative stress that may contribute to fatigue and impaired athletic performance [14]. Curcumin, through its antioxidant and anti-inflammatory actions, may exert a protective effect on cell membranes and secondary muscle damage processes [15]. However, ROS also act as biological messengers that regulate gene transcription and physiological adaptation to training [16,17]. Long-term antioxidant supplementation has been suggested to reduce some exercise-induced adaptations, including performance-related [18,19] and health-related benefits [20]. These adaptations partly aim to enhance endogenous antioxidant defenses, which explains why individuals who regularly exercise show increased tolerance to exercise-induced oxidative stress [21,22,23]. Nevertheless, previous evidence has not clearly shown that curcumin supplementation impairs training adaptations [24]. Given this dual role of reactive oxygen species in both fatigue-related processes and training adaptation, the practical use of curcumin in sport recovery requires a careful balance between attenuating excessive oxidative or inflammatory responses and avoiding interference with physiological adaptation [25].

Several systematic reviews and meta-analyses have evaluated curcumin supplementation in exercise-related outcomes, including inflammation, oxidative stress, muscle damage, soreness, and physical performance [24,26,27,28,29,30,31]. Nanavati et al. [24] reported that curcumin doses ranging from 90 to 5000 mg/day may reduce perceived muscle soreness, enhance antioxidant capacity, and lower CK levels, particularly when administered near exercise. Vasile et al. [26] concluded that curcumin may alleviate delayed-onset muscle soreness following exercise-induced damage and may improve antioxidant and inflammatory biomarkers. Meta-analyses by Fang and Nasir [27] and Liu et al. [28] further reported reductions in CK, interleukin-6 (IL-6), and soreness, and improvements in range of motion, while highlighting the influence of dose and timing. Suhett et al. [29] and Fernández-Lázaro et al. [30] also supported the potential role of curcumin in sports and physical exercise, whereas Oxley and Peart [31] reported less consistent effects on functional strength, delayed-onset muscle soreness, and inflammatory markers.

Although these reviews provide relevant evidence, most have either focused on exercise-induced muscle damage biomarkers or included protocols specifically designed to induce substantial damage, such as downhill running or isolated unaccustomed eccentric contractions [24,26,27,28,29,30,31]. Such models are useful mechanistically but may produce levels of muscle disruption that exceed those commonly observed in training or sport practice, limiting direct application to athletes [32]. In addition, prior reviews have rarely considered dietary control, exercise-load monitoring, participant training status, formulation type, and bioavailability verification together.

Beyond excluding artificial muscle-damage models, the present review also differs from previous evidence syntheses by jointly considering the ecological validity of the exercise context, participant training status, supplementation timing, formulation type, dietary control, training-load monitoring, and the practical transferability of outcomes. This distinction is relevant because findings derived from laboratory-based or mechanistic protocols may not translate directly to sport practice, where recovery responses are influenced by training background, accumulated load, competitive demands, and the relevance of functional or performance-related outcomes. Consequently, classifying the real-world sport applicability of the included studies allows the evidence to be interpreted not only according to statistical findings but also according to its practical relevance for athletes and practitioners.

Therefore, the aim of the present systematic review was to evaluate the effects of curcumin supplementation on exercise-related recovery outcomes, including muscle damage, inflammation, oxidative stress, subjective recovery, fatigue, and physical performance, in healthy active individuals and athletes. A secondary aim was to examine the applicability of the available evidence to real-world sport settings by distinguishing studies conducted in athletes under match-play, competition-like, race-based, field-based, or applied training contexts from studies conducted in recreationally active, healthy, or laboratory-based populations.

## 2. Materials and Methods

### 2.1. Search Strategy

A comprehensive literature search was conducted in PubMed, Scopus, Web of Science, SPORTDiscus, and Cochrane Library/CENTRAL from 2012 until June 2026. Searches were performed in title and abstract fields using terms related to curcumin, exercise recovery, muscle damage, oxidative stress, inflammation, performance, and sport or exercise contexts. The complete electronic search strategies for each database, including the number of records retrieved, are provided in Supplementary Table S1. Only articles published in English or Spanish were considered. This systematic review was reported according to the Preferred Reporting Items for Systematic Reviews and Meta-Analyses (PRISMA) 2020 statement [33] and was prospectively registered in PROSPERO under the ID CRD420251229038. The completed PRISMA 2020 checklist is provided in Supplementary Table S2.

### 2.2. Eligibility Criteria and Study Selection

The Population, Intervention, Comparator, Outcomes, and Study design (PICOS) criteria used to determine eligibility are presented in Table 1. Studies were eligible when they included healthy human participants; evaluated oral curcumin, curcuminoids, curcuma-derived preparations with a specified curcumin dose, or curcumin combined only with bioavailability enhancers; and assessed at least one exercise-related recovery, fatigue, performance, inflammatory, oxidative stress, or muscle-damage outcome.

Studies were excluded if they: (1) used curcumin in combination with additional bioactive compounds not included exclusively to improve curcumin bioavailability; (2) did not specify the quantity of curcumin or curcuminoids administered; (3) were review articles, animal studies, in vitro studies, non-randomized trials or lacked a double-blind design; (4) included non-healthy or clinical populations; (5) focused on sarcopenia, frailty, or disease-related muscle loss rather than exercise-related responses; (6) used protocols specifically designed to induce maximal artificial muscle damage, such as downhill running or isolated unaccustomed eccentric contractions, when these protocols lacked ecological relevance to training or sport practice; or (7) did not assess muscle damage, inflammation, oxidative stress, subjective recovery, fatigue, or physical performance outcomes.

Artificial muscle-damage protocols were operationally defined as isolated, unaccustomed, or deliberately damage-inducing eccentric protocols designed primarily to maximize muscle damage rather than reflect habitual training or sport practice. Examples included isolated eccentric elbow-flexor protocols or downhill running protocols specifically designed to induce substantial muscle damage. In contrast, laboratory-based protocols were considered eligible when they involved whole-body running, cycling, resistance exercise, high-intensity interval training, treadmill exercise, match-play, competition-like, or structured training contexts relevant to exercise recovery. Therefore, laboratory-based studies were not excluded a priori; instead, they were included when they represented an exercise or sport-relevant recovery context and were subsequently classified as having high, moderate, or low/indirect real-world sport applicability according to their transferability to applied sport practice.

All records retrieved from the database searches were imported into EndNote Web, where duplicate records were identified and removed before screening. Two reviewers (J.L.-G. and F.J.M.-N.) independently screened titles and abstracts according to the predefined PICOS criteria. Full-text articles were then assessed independently by the same two reviewers to determine final eligibility. Any disagreements during title/abstract screening or full-text assessment were resolved through discussion and, when necessary, by consultation with a third reviewer (D.V.-M.). Inter-rater agreement for study selection was high (Cohen’s κ = 0.89). Reasons for exclusion at the full-text stage were recorded and are presented in Supplementary Table S3.

### 2.3. Data Extraction

Data extraction was performed independently by two reviewers (J.L.-G. and F.J.M.-N.) using a predefined data extraction form. Any discrepancies were resolved by discussion and, when required, by consultation with a third reviewer (D.V.-M.). The following information was extracted from each included study: first author and year of publication, country, study design, sample size, participant characteristics, sex distribution, age, training status, sport or exercise context, supplementation duration, daily curcumin dose, formulation type, use of bioavailability enhancers, timing of ingestion, comparator, exercise or sport protocol, dietary control, exercise-load monitoring, outcomes assessed, time points of assessment, adverse events when reported, and main findings.

Outcome variables were grouped into the following domains: muscle damage, inflammation, oxidative stress, subjective recovery or soreness, fatigue, and physical performance. When available, details on commercial curcumin formulations, verification of supplement composition, and assessment of circulating curcumin exposure were also extracted. Training status was classified according to the athlete classification framework described below in order to distinguish between healthy active individuals, recreationally active participants, amateur or developmental athletes, and highly trained or professional athletes.

In addition, the real-world sport applicability of each included study was classified as high, moderate, or low. This classification was based on four criteria: participant training status, ecological validity of the exercise protocol, relevance of the outcomes to sport recovery or performance, and transferability of the supplementation strategy to applied sport settings. High applicability was assigned to studies conducted in athletes or sport-specific populations under match-play, competition-like, race-based, or field-based sport contexts, with outcomes directly related to recovery or performance. Moderate applicability was assigned to studies involving athletic, recreationally active, or trained populations assessed under structured training interventions, laboratory-based protocols with partial sport transferability, or field-relevant exercise challenges. Low applicability was assigned to studies conducted mainly in non-athlete, healthy active, or highly controlled laboratory-based populations, particularly when outcomes were predominantly biochemical and had limited direct transferability to applied sport practice. The study-level classification and rationale are provided in Supplementary Table S6. Applicability classification was performed independently by two reviewers (J.L.-G and F.J.M.-N.). Disagreements were resolved by consensus and, when necessary, by consultation with a third reviewer (D.V.-M.).

When available, effect sizes, confidence intervals, or standardized effect estimates were extracted or considered for interpretation. However, a formal effect-size synthesis was not performed because outcomes, biomarkers, time points, exercise protocols, and statistical reporting were highly heterogeneous across trials. Therefore, findings were synthesized narratively and by direction of effect, while the certainty of evidence was evaluated using the Grading of Recommendations Assessment, Development and Evaluation (GRADE).

A favorable effect was defined as a statistically significant between-group difference or group-by-time interaction in favor of curcumin for at least one prespecified outcome within a given domain. These classifications were used only as a descriptive direction-of-effect summary and should not be interpreted as evidence that all outcomes or biomarkers within that domain improved consistently.

### 2.4. Quality Assessment

The methodological quality of the included studies was evaluated using the Physiotherapy Evidence Database (PEDro) scale [34], a validated tool commonly used to assess the methodological quality of randomized clinical trials. The PEDro scale was selected because it is widely used in physiotherapy, exercise science, sports medicine, and rehabilitation research and is particularly suitable for randomized trials involving exercise-based protocols, performance-related outcomes, and applied sport or recovery settings. In the present review, PEDro was used to appraise the methodological quality, whereas Cochrane RoB 2 was applied as a supplementary domain-based risk-of-bias assessment. This approach was considered appropriate given the applied sport context of the included studies and the frequent use of exercise performance, recovery, soreness, and biomarker-based outcomes. Two independent reviewers assessed each study, and disagreements were resolved through discussion or, when necessary, by consultation with a third reviewer.

The PEDro scale comprises 11 items, although the first item, related to eligibility criteria, is not included in the total score. The remaining items assess key elements of internal validity and statistical reporting, including random allocation, allocation concealment, baseline comparability, blinding of participants, blinding of personnel administering the intervention, blinding of outcome assessors, adequate follow-up, intention-to-treat analysis, between-group comparisons, and reporting of point estimates and measures of variability. Therefore, the final PEDro score ranges from 0 to 10, with higher scores indicating greater methodological quality.

The PEDro items were as follows: (1) eligibility criteria were specified; (2) subjects were randomly allocated to groups; (3) allocation was concealed; (4) groups were similar at baseline regarding the most important prognostic indicators; (5) subjects were blinded; (6) personnel administering the intervention were blinded; (7) assessors measuring at least one key outcome were blinded; (8) measures of at least one key outcome were obtained from more than 85% of the subjects initially allocated to groups; (9) all subjects for whom outcome measures were available received the treatment or control condition as allocated, or, when this was not the case, data for at least one key outcome were analyzed by intention to treat; (10) between-group statistical comparisons were reported for at least one key outcome; and (11) the study provided both point estimates and measures of variability.

As a supplementary domain-based assessment of risk of bias, the Cochrane Risk of Bias 2 tool (RoB 2) was also applied to the included randomized trials. Parallel-group trials were assessed using the standard RoB 2 tool, whereas crossover trials were assessed using the RoB 2 extension for crossover trials, which includes an additional domain addressing bias arising from period and carryover effects [35]. Judgements were made at the domain level and overall as “low risk of bias”, “some concerns”, or “high risk of bias”. The effect of interest was selected according to the analysis reported in each trial, distinguishing between intention-to-treat and per-protocol effects when appropriate. Two reviewers (J.L.-G. and D.V.-M.) independently assessed risk of bias, and disagreements were resolved by discussion or, when necessary, by consultation with a third reviewer.

The certainty of evidence was assessed using the GRADE approach with GRADEpro GDT (McMaster University and Evidence Prime Inc., Hamilton, ON, Canada) [36,37,38]. Because meta-analysis was not conducted owing to substantial clinical and methodological heterogeneity across studies, a narrative Summary of Findings table was developed at the outcome-domain level rather than for pooled effect estimates. The assessed domains were oxidative stress, muscle damage, inflammation, subjective recovery/soreness/fatigue, and physical or athletic performance. Randomized trials were initially considered high-certainty evidence and were downgraded, when appropriate, for risk of bias, inconsistency, indirectness, imprecision, and publication bias.

## 3. Results

### 3.1. Selection of the Studies

**Figure 1:**
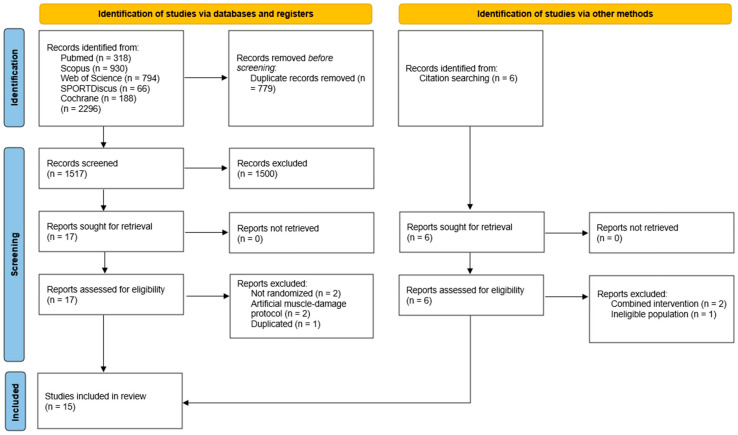
Preferred Reporting Items for Systematic Reviews and Meta-Analyses (PRISMA) 2020 flow diagram of the study selection process.

The literature search identified a total of 2296 records (PubMed: 318; Web of Science: 794; Scopus: 930; SPORTDiscus: 66; Cochrane Library/CENTRAL: 188). After removing 779 duplicates, 1517 records were screened by title and abstract. Of these, 1500 were excluded. Seventeen full-text reports from database searching were assessed for eligibility. In addition, six relevant reports were identified through citation searching, resulting in 23 full-text reports assessed for eligibility. Eight reports were excluded after full-text assessment or reassessment: non-randomized design (*n* = 2), artificial muscle-damage protocol (*n* = 2), duplicate report (*n* = 1), combined intervention (*n* = 2), and ineligible population/outcome (*n* = 1). Finally, 15 randomized double-blind placebo-controlled trials [39,40,41,42,43,44,45,46,47,48,49,50,51,52,53] were included in the systematic review (Figure 1). Full-text exclusion reasons are presented in Supplementary Table S3.

### 3.2. Methodological Quality Assessment

The methodological quality of the included randomized placebo-controlled trials was assessed using the PEDro scale (Table 2). Overall, the included studies showed moderate methodological quality, although methodological rigor varied across trials. Most studies reported random allocation and baseline comparability between groups, whereas allocation concealment, blinding of all relevant parties, intention-to-treat analysis, and complete follow-up were less consistently reported. The main methodological limitations identified across studies were small sample sizes, incomplete reporting of allocation procedures, limited dietary and exercise-load control, and scarce verification of circulating curcumin exposure or supplement composition.

The RoB 2 assessment reported in Supplementary Figures S1–S4 showed that several trials presented at least some risk-of-bias concerns. The most frequent concerns were related to incomplete reporting of the randomization process, selection of the reported result, missing outcome data in some trials, and, in crossover studies, potential period or carryover effects. Overall judgements varied across studies and according to the effect of interest assessed. The complete RoB 2 domain-level assessments for parallel and crossover trials, including intention-to-treat and per-protocol effects where applicable, are presented in Supplementary Figures S1–S4.

### 3.3. Certainty of Evidence

The GRADE assessment indicated low certainty of evidence for oxidative-stress outcomes and very low certainty of evidence for muscle damage, inflammation, subjective recovery/soreness/fatigue, and physical or athletic performance. The main reasons for downgrading were risk-of-bias concerns, inconsistency across studies and biomarkers, indirectness for subjective recovery and performance-related outcomes, and imprecision due to small sample sizes and the absence of pooled effect estimates. The complete narrative Summary of Findings table is provided in Supplementary Table S5.

### 3.4. Characteristics of Included Studies

Participants were categorized according to the athlete development framework proposed by McKay et al. [54], which stratifies individuals based on training volume, competitive level, and sport-specific development. Accordingly, four broad categories were used in the present review: tier 0, healthy untrained or non-athlete participants not engaged in structured exercise; tier 1, recreationally active individuals or physically active non-athletes performing regular exercise without systematic competition-level training; tier 2, amateur or developmental athletes engaged in structured training programs and local or amateur-level competition; and tier 3, highly trained or professional athletes with high-volume structured training and regular participation in high-level competition. This classification allowed consistent comparison of curcumin’s effects across populations with different training backgrounds.

The 15 articles included in this review encompassed participants with varying training backgrounds, ranging from healthy active adults and recreationally active individuals to amateur athletes and highly trained football players. Several studies were conducted in sport-specific or competition-like settings. These included professional or collegiate football/soccer match play [39,52], futsal match play [43], simulated taekwondo competitions [42], half-marathon running [41], and football athletes receiving a Curcuma-derived intervention [50]. Other studies were conducted in controlled exercise or training settings, including endurance cycling [51], treadmill-based endurance exercise [53], 14 km running [49], resistance exercise to exhaustion [46], cycling combined with a dual stress challenge [47], and high-intensity interval training [44]. Finally, some trials evaluated curcumin supplementation during longer intervention periods in healthy active adults or amateur endurance athletes, including Bańkowski et al. [40], Li et al. [45], and Salehi et al. [48].

The contextual and methodological characteristics of the included studies are presented in Table 3. Dietary control, exercise-load monitoring, and verification of curcumin exposure or supplement composition were heterogeneous across trials. Some studies implemented standardized diets, dietary records, objective match-load monitoring, or standardized exercise protocols, whereas others provided limited or unclear control of these variables. These aspects should be considered when interpreting the consistency and applicability of the findings.

Based on this classification, six studies showed high direct applicability to real-world sport settings [39,41,42,43,50,52], four studies showed moderate applicability [40,44,49,51], and five studies provided low or indirect applicability [45,46,47,48,53]. This distinction was considered important when interpreting the practical relevance of curcumin supplementation for athletes and sport practitioners.

### 3.5. Bioavailability and Formulation Considerations

Several formulation technologies have been developed to overcome curcumin’s poor bioavailability by improving its solubility, stability, and intestinal absorption. These strategies mainly rely on enhancing the dispersion of curcumin in aqueous media, preventing its rapid conjugation, or facilitating its transport through lipid-based carriers. Table 4 summarizes the principal formulation approaches, their compositional characteristics, and the reported fold increases in systemic exposure/area under the curve compared with unformulated curcumin. Importantly, the comparison also specifies whether bioavailability values were derived from hydrolyzed plasma samples, reflecting total curcuminoids including conjugated metabolites, or from non-hydrolyzed samples, reflecting free curcumin, as this methodological distinction substantially influences the interpretation of pharmacokinetic outcomes.

The included studies used heterogeneous curcumin preparations. Some studies administered standard curcumin or curcuma-derived preparations [41,42,49,50]. Curcumin combined with piperine was evaluated in the futsal study by Juniarsyah et al. [43], and this combination was retained because piperine was used as a bioavailability enhancer. Enhanced or optimized formulations were used in several studies, including colloidal or nanoparticle dispersions, solid lipid formulations, phytosome-based preparations, micellar or dispersion delivery systems, and other commercial bioavailability-enhanced products. Abbott et al. [39] evaluated an acute enhanced-bioavailability curcumin intervention in a football match-play context, reporting improvements in soreness and functional recovery, although biological muscle-damage biomarkers were not assessed. The commercial formulations identified across the included studies are summarized in Supplementary Table S4, which links each product to its formulation category, proposed bioavailability-enhancement strategy, curcumin dose, and available information on supplement verification or circulating curcumin assessment. Thus, Table 4 provides the pharmacokinetic rationale for the main formulation technologies, whereas Supplementary Table S4 shows how these technologies were represented across the included trials.

Overall, no clear pattern of superiority was observed for any specific formulation type. Favorable or partially favorable findings were reported across both standard and enhanced-bioavailability preparations, whereas several trials using optimized formulations also reported null or mixed effects. Therefore, formulation type may be an important moderator of response, but the current evidence does not allow its independent contribution to be isolated because formulation is confounded by dose, timing, exercise protocol, training status, outcome selection, and limited verification of circulating curcumin exposure.

### 3.6. Timing of Supplementation

Studies were classified according to the duration and timing of curcumin intake. Acute or peri-exercise interventions were defined as protocols in which curcumin was administered shortly before and/or after a single exercise bout, competition, or short recovery period. Chronic interventions involved continuous supplementation for more than five days, either during a training period or before a defined exercise or competition challenge.

When stratified by supplementation pattern (Table 5), the included articles were distributed between acute/peri-exercise protocols and longer supplementation protocols. Acute or peri-exercise protocols included studies in which curcumin was administered around a specific exercise or sport challenge. This category included the football match-play studies by Abbott et al. [39] and Tanabe et al. [52], the simulated taekwondo competition study by Ghojazadeh et al. [42], the resistance exercise recovery study by Mallard et al. [46], the endurance cycling study by Sciberras et al. [51], the treadmill exercise study by Takahashi et al. [53], and the cycling dual stress challenge study by McAllister et al. [47].

Longer supplementation protocols included studies administering curcumin for periods ranging from 7 days to 8 weeks. These included the futsal match-play study by Juniarsyah et al. [43], the half-marathon study by Faria et al. [41], the 14 km running study by Nakhostin Roohi et al. [49], the amateur long-distance runner study by Bańkowski et al. [40], the high-intensity interval training study by Kisiolek et al. [44], the healthy active women study by Salehi et al. [48], the healthy adult oxidative stress trial by Li et al. [45], and the football athlete study by Rosidi et al. [50].

Among acute or peri-exercise interventions, findings were mixed. Abbott et al. [39] reported that curcumin attenuated delayed-onset muscle soreness and muscle function deficits after a soccer match in professional male players. In contrast, Tanabe et al. [52] observed no significant benefit of curcumin on muscle soreness, jump performance, C-reactive protein, creatine kinase, or urinary titin after a soccer match in collegiate players. Ghojazadeh et al. [42] reported favorable effects on muscle damage and oxidative stress markers after successive simulated taekwondo competitions, whereas Mallard et al. [46] observed reduced delayed-onset muscle soreness, lower thigh circumference, and lower post-exercise lactate after resistance exercise to exhaustion. Although interleukin-10 was higher at 24 h, interleukin-6 was also higher at selected post-exercise time points, and no significant between-group differences were observed for creatine kinase, lactate dehydrogenase, myoglobin, high-sensitivity C-reactive protein, or tumor necrosis factor-alpha. Sciberras et al. [51] and Takahashi et al. [53] focused mainly on inflammatory and oxidative stress responses after endurance-type exercise, with variable effects across markers. McAllister et al. [47] did not report a clear treatment effect on oxidative stress markers following a combined mental and physical stress challenge.

Chronic or longer supplementation protocols were also heterogeneous. Juniarsyah et al. [43] reported lower creatine kinase, aspartate aminotransferase, and alanine aminotransferase responses and improvements in CMJ and 20-m sprint performance after two consecutive futsal matches. Faria et al. [41] found that turmeric extract supplementation increased interleukin-10 and reduced myoglobin after a half-marathon race, while Nakhostin Roohi et al. [49] reported improvements in selected oxidative stress markers after a 14 km run. Bańkowski et al. [40] did not find significant changes in inflammatory markers in amateur long-distance runners, whereas Kisiolek et al. [44] reported that optimized curcumin did not impair performance adaptations during high-intensity interval training. Salehi et al. [48] reported improvements in C-reactive protein, lactate dehydrogenase, malondialdehyde, and maximal oxygen uptake (VO_2_max) in healthy women with moderate physical activity, and Li et al. [45] reported improvements in selected oxidative stress indices after 8 weeks of amorphous curcumin supplementation. Rosidi et al. [50] reported reductions in malondialdehyde in football athletes receiving Curcuma-derived curcumin.

### 3.7. Outcome Domains and Narrative Synthesis Structure

Table 6 summarizes the main characteristics of the 15 randomized double-blind placebo-controlled trials included in the present review. The studies differed considerably in terms of participant characteristics, supplementation duration, administered dose, formulation type, and exercise protocol. Supplementation periods ranged from acute or peri-exercise interventions to chronic protocols lasting up to 8 weeks, with curcumin doses varying widely across studies and preparations. Curcumin was administered as standard curcumin or turmeric-derived extracts in combination with piperine or as enhanced-bioavailability formulations designed to improve systemic exposure.

The exercise protocols also varied widely, including football and futsal match play, simulated taekwondo competitions, half-marathon running, 14 km running, endurance cycling, treadmill-based exercise, resistance exercise to exhaustion, high-intensity interval training, and laboratory-based exercise challenges. The main outcomes assessed across studies included performance and functional recovery, muscle damage markers, inflammatory biomarkers, oxidative stress and antioxidant markers, subjective recovery, soreness, and fatigue-related responses. Overall, Table 6 provides an overview of these methodological differences and summarizes the main outcomes related to performance, inflammation, oxidative stress, muscle damage, and recovery.

### 3.8. Training Status and Context of Evidence

According to the athlete development framework proposed by McKay et al. [54], the included studies covered a broad range of training backgrounds, from healthy untrained or moderately active non-athletes to recreationally active participants, amateur/development-level athletes, and highly trained or professional players. As summarized in Table 7, most evidence came from recreationally active individuals and amateur or development-level athletes, whereas evidence in highly trained or professional athletes remained limited. This distribution highlights the heterogeneity of the populations investigated and supports the need to interpret curcumin’s effects according to training background, exercise context, and physiological conditioning.

These counts indicate the presence of at least one favorable finding within each domain and should not be interpreted as showing that all biomarkers, time points, or outcomes within that domain improved consistently. Statistical significance was interpreted separately from practical relevance and certainty of evidence. Because many included studies had small sample sizes and assessed multiple biomarkers or time points, isolated significant findings were not considered sufficient to infer clinically or practically meaningful recovery benefits. Practical relevance was interpreted cautiously in relation to the type of outcome assessed, the ecological validity of the exercise protocol, and the certainty of evidence according to GRADE.

In healthy untrained non-athletes, evidence regarding curcumin supplementation is limited to a single study assessing oxidative stress and performance outcomes following a cardiorespiratory endurance test [45]. Therefore, whether these findings translate to trained athletes remains uncertain. In recreationally active individuals, curcumin was investigated across resistance exercise, endurance exercise, high-intensity interval training, and controlled laboratory-based protocols. Mallard et al. [46] reported improvements in delayed-onset muscle soreness and post-exercise lactate accumulation after resistance exercise to exhaustion. Among recreationally active individuals, the included studies were mainly conducted under laboratory-based endurance, cycling, resistance-exercise, dual-stress, or high-intensity interval training conditions. Findings were heterogeneous, with favorable effects reported for selected soreness, oxidative-stress, and lactate-related outcomes but inconsistent effects for inflammatory biomarkers and performance-related measures.

In amateur or development-level athletes, evidence came from more sport-specific contexts, including futsal, football/soccer, half-marathon running, endurance running, and field-based running tests. Favorable findings were reported for selected muscle-damage or oxidative-stress outcomes in some studies, whereas other trials showed no clear improvements in inflammatory, soreness, jump-performance, or broader recovery-related outcomes. Overall, findings in this subgroup were mixed and appeared to vary according to sport context, outcome domain, and assessment timing.

Evidence in highly trained or professional athletes was limited to two studies conducted on professional soccer players and trained taekwondo athletes [39,42]. These studies reported favorable effects on selected soreness, muscle-function, muscle-damage, and oxidative-stress outcomes, although inflammatory findings were less consistent. Given the small number of studies in high-performance populations, conclusions for elite athletes remain preliminary.

## 4. Discussion

This systematic review sought to determine whether curcumin supplementation supports post-exercise recovery and performance in healthy active individuals and athletes exposed to exercise, training, or sport-related contexts. A total of 15 randomized double-blind placebo-controlled trials were included encompassing exercise models ranging from controlled laboratory-based protocols and structured training sessions to competitive or simulated match scenarios. Across studies, curcumin demonstrated potential beneficial effects in multiple domains relevant to recovery, with the most consistent findings observed in markers of oxidative stress, followed by selected muscle damage and functional or subjective recovery/fatigue-related outcomes, whereas effects on inflammation and performance-related outcomes were more variable across studies.

The heterogeneity of findings across studies likely reflects the combined influence of participant training status, exercise modality, supplementation regimen, formulation type, and outcome assessment. Recreationally active individuals may respond differently from trained or professional athletes because repeated training exposure increases tolerance to exercise-induced oxidative and inflammatory stress. Exercise protocols also varied markedly, ranging from field-based match play and endurance races to laboratory cycling, treadmill exercise, resistance exercise, high-intensity interval training, and dual-stress challenges. In addition, supplementation duration ranged from acute peri-exercise dosing to several weeks of intake, while formulations differed substantially in expected bioavailability. Finally, sampling time points and biomarker panels were not standardized, making it difficult to determine whether null findings reflected absence of effect, inadequate timing of assessment, insufficient statistical power, or insufficient biological exposure.

### 4.1. Applicability to Real-World Sport Settings

A central contribution of the present review is the distinction between general exercise-based evidence and findings that can be directly transferred to applied sport practice. Although all included studies were randomized double-blind placebo-controlled trials assessing curcumin in relation to exercise-related outcomes, their real-world applicability differed substantially. Six studies were conducted in athlete-based match-play, competition-like, race-based, or field-based contexts and therefore provide the most directly applicable evidence for athletes and practitioners [39,41,42,43,50,52]. Four studies were classified as having moderate applicability because they involved athletic, recreationally active, or trained populations assessed partly under laboratory conditions, structured training interventions, or field-relevant exercise challenges [40,44,49,51]. The remaining five studies provided low or indirect applicability because they were conducted mainly in healthy, recreationally active, or laboratory-based populations or because the exercise protocol and outcomes had limited direct transferability to applied sport recovery or performance [45,46,47,48,53]. These studies remain valuable for understanding potential mechanisms, particularly oxidative stress and inflammatory responses, but their transferability to competitive athletes should be interpreted with caution.

When the evidence is restricted to higher-applicability sport contexts, curcumin appears most promising for selected markers of muscle damage, oxidative stress, and subjective recovery after repeated or high-intensity sport demands. However, the number of trials conducted in athletes under ecologically valid training or competition conditions remains small, and the available studies differ in sport modality, competitive level, dose, formulation, supplementation timing, and outcomes assessed. Therefore, the present findings support curcumin as a potentially useful recovery strategy in selected applied sport contexts, particularly when repeated high-intensity training or competition demands are present, but they do not yet allow universal recommendations across all athlete populations, competitive levels, or sport modalities.

### 4.2. Interpretation of Findings and Comparison with Previous Evidence

Previous systematic reviews evaluating curcumin in exercise settings have primarily focused on protocols intentionally designed to induce muscle damage, including eccentric loading and downhill running models [11,63,64,65,66,67,68,69,70,71,72]. However, such protocols may produce levels of muscle disruption that exceed those typically observed in applied sport scenarios, limiting the ecological validity of their findings and their applicability to athletes [32]. In contrast, the present review focused on exercise- and sport-related contexts in healthy active individuals and athletes, while excluding artificial protocols specifically designed to induce substantial muscle damage. This approach allows a more practice-oriented appraisal of curcumin’s potential relevance for applied recovery strategies. Furthermore, this review stratified outcomes according to athlete training status using the McKay et al. [54] classification framework, providing a differentiated interpretation of curcumin’s effects across levels of sporting proficiency and physiological adaptation. This is particularly relevant because findings derived from untrained or recreational populations may not be directly transferable to highly trained or elite athletes, who typically display greater resilience to exercise-induced inflammation and oxidative stress [32]. Another novel contribution is the systematic assessment of dietary control and training-load monitoring across trials, factors that have been largely overlooked in prior evidence syntheses despite their known impact on inflammation, oxidative responses, and recovery dynamics [73]. Together, these methodological distinctions position the present review as a complementary and practice-oriented analysis relative to earlier work.

Variation in the direction and magnitude of effects across studies appeared to be influenced by training status, exercise context, and ecological validity. Most of the available evidence came from recreationally active individuals and amateur or development-level athletes, whereas data in highly trained or professional athletes remained scarce. Among the studies with high direct applicability to real-world sport settings, favorable findings were observed mainly for selected markers of muscle damage, oxidative stress, and subjective recovery, while effects on inflammatory and performance-related outcomes were less consistent. This variability may reflect differences in sport modality, competitive level, external load, recovery window, biomarker selection, and timing of assessment. Therefore, although the applied sport evidence is promising, the limited number of trials conducted in ecologically valid athlete populations precludes firm conclusions regarding the efficacy of curcumin at higher performance levels.

Although some excluded studies have examined curcumin supplementation in competitive or trained athletes, these were not used to support the main interpretation because of relevant methodological limitations, including non-randomized designs, absence of placebo control or blinding, very small sample sizes, and limited dietary or training-load monitoring. These studies may provide contextual information, but they reinforce rather than resolve the need for rigorously controlled trials in genuinely high-performance sport settings.

Differences in supplementation duration did not yield a uniform pattern across outcomes. Oxidative stress-related markers appeared responsive in both acute/peri-exercise and longer-term protocols, whereas performance-related outcomes were less consistent, partly because studies assessed different constructs, including match-related functional recovery, time-trial performance, VO_2_max, lactate responses, and fatigue-related indices. Some null findings may also reflect insufficient physiological or functional perturbation induced by the exercise protocol, inadequate timing of outcome assessment, or limited statistical power. Overall, supplementation duration may modulate responsiveness in a domain-specific manner, but the available evidence does not support a generalizable effect across all recovery outcomes.

Further research directly comparing acute and chronic regimens is required to clarify whether specific physiological pathways respond preferentially to repeated or peri-exercise curcumin intake. The limited number of acute trials included in this review did not allow firm conclusions regarding the optimal timing of curcumin ingestion, as the distribution of studies across pre-, post-, and combined dosing protocols was small and findings were heterogeneous. When considered alongside the broader literature, the available evidence suggests a more consistent pattern. Several previous reviews have reported that post-exercise ingestion may be more effective, particularly for attenuating muscle-damage and inflammatory responses. For example, Vasile et al. [26] highlighted post-exercise supplementation as the most favorable strategy, and Liu et al. [28] concluded that immediate post-exercise intake was more effective at reducing CK and IL-6 concentrations, whereas pre-exercise ingestion may offer benefits for muscle soreness or range of motion in certain contexts.

Evidence from excluded eccentric muscle-damage models suggests that post-exercise curcumin ingestion may produce more pronounced effects on soreness, range of motion, creatine kinase, and functional recovery than pre-exercise ingestion [63,64]. However, because these studies used artificial damage-inducing protocols, their findings should be interpreted as mechanistic context rather than direct evidence for applied sport recovery. Within the trials included in the present review, the small number of studies across pre-, post-, and combined dosing strategies prevents firm conclusions regarding optimal timing.

Formulation type may also influence the physiological responses to curcumin, although the available evidence does not support a consistent superiority of any specific delivery system. Curcumin exhibits inherently low bioavailability due to poor solubility, extensive first-pass metabolism, and rapid systemic elimination, and numerous strategies—including piperine co-administration, phospholipid complexes, micellar systems, and colloidal or nanoparticle dispersions—have been developed to enhance absorption. Previous evidence syntheses have often recommended the use of bioavailability-enhanced formulations; for instance, an umbrella review on curcumin for weight- and adiposity-related outcomes [74] reported greater reductions in body weight, BMI, and waist circumference with enhanced formulations compared with standard preparations, and a large evidence synthesis screening more than 4000 records [75] similarly advocated prioritizing formulations with demonstrated pharmacokinetic advantages in clinical settings.

However, findings from the present review do not fully align with the assumption that enhanced bioavailability necessarily produces superior recovery-related effects. Favorable findings were observed across both standard curcumin or curcuma-derived preparations and enhanced-bioavailability formulations, whereas several trials using optimized formulations also reported null or mixed effects. This observation is consistent with a recent meta-analysis in individuals with prediabetes or type 2 diabetes [76], in which unformulated curcumin produced larger effects on malondialdehyde, reduced glutathione, and CRP than piperine-containing or enhanced-bioavailability formulations, the latter of which showed no significant impact on CRP. The study-level information summarized in Supplementary Table S4 further supports this interpretation, as enhanced-bioavailability products differed markedly in composition, curcumin dose, delivery technology, supplement verification, and assessment of circulating curcumin exposure. Accordingly, enhanced bioavailability should be considered a plausible but unconfirmed moderator of efficacy, rather than sufficient evidence of practical superiority in sport recovery contexts.

Dosing considerations also warrant attention. Across the included trials, curcumin intakes varied substantially, ranging from low-dose enhanced formulations to gram-level intakes in acute or peri-exercise protocols and from approximately 90 mg/day to 2000 mg/day in longer-term interventions lasting up to 8 weeks. These intakes frequently exceeded the acceptable daily intake (ADI) of 3 mg·kg^−1^·day^−1^, equivalent to approximately 210 mg/day for a 70 kg adult, established by the European Food Safety Authority (EFSA) for curcumin when used as a food additive [77]. However, the EFSA ADI was derived within the context of food additive exposure, not supplementation, and numerous clinical trials have administered gram-level doses of curcumin for weeks to months without reporting serious adverse events or hepatotoxicity under controlled conditions [78,79,80,81,82].

Interpretation of formulation-related effects is further limited by methodological variability in pharmacokinetic assessment. Differences in the quantification of free versus total curcuminoids, together with the wide diversity of curcumin formulations used across studies, make direct comparisons difficult. Therefore, well-controlled trials directly comparing unformulated and enhanced-delivery preparations, using standardized pharmacokinetic methods, are needed to clarify whether formulation type meaningfully modulates efficacy in exercise- and sport-specific contexts.

A further methodological limitation concerns the verification of curcumin exposure and supplement composition. Only a minority of trials quantified circulating curcumin or its metabolites, and few analytically confirmed supplement content using high-performance liquid chromatography or other analytical methods. This is relevant given curcumin’s susceptibility to degradation under heat, light, humidity, or suboptimal storage conditions, which may reduce the concentration of bioactive compounds [83,84]. Future studies should incorporate standardized plasma measurements, ideally distinguishing free from conjugated metabolites, and analytical verification of supplement composition to strengthen interpretation of dose–exposure–response relationships.

Dietary intake and training-load exposure may also have contributed to the heterogeneity of findings. Energy availability, carbohydrate and protein intake, antioxidant-rich foods, omega-3 fatty acids, and day-to-day variations in training load can influence inflammatory, oxidative, performance-related, and recovery processes [85,86,87,88]. However, only a minority of studies implemented rigorous dietary or load control, with most relying on partial recalls, brief logs, or no monitoring. This limits attribution of effects to curcumin itself, since uncontrolled variation in diet, previous exercise, or accumulated fatigue could obscure or inflate supplementation effects.

The patterns observed across studies are nevertheless compatible with curcumin’s established actions on inflammatory and redox pathways activated during exercise. By modulating cytokine signaling and supporting endogenous antioxidant responses, curcumin may reduce secondary muscle damage and facilitate the recovery of muscle function following strenuous activity [89,90]. However, evidence regarding chronic supplementation and training adaptation remains limited. While some studies reported less favorable or attenuated performance changes with curcumin [41,44], others observed improvements in aerobic capacity [48]. Overall, current evidence does not provide clear evidence that curcumin impairs training adaptations; however, this conclusion should be interpreted cautiously because the available trials were few, heterogeneous, and not primarily designed to assess long-term training adaptations.

A key practical issue is whether changes in oxidative stress or inflammatory biomarkers translate into meaningful improvements in recovery, training readiness, or athletic performance. In the present review, biomarker-based findings were generally more favorable for oxidative stress than for inflammation, muscle damage, or performance outcomes. However, improvements in markers such as malondialdehyde, total antioxidant capacity, total oxidant status, or oxidative stress index did not consistently coincide with improvements in soreness, neuromuscular function, training capacity, or sport-specific performance. This limits the practical interpretation of isolated biochemical changes and suggests that future studies should combine biomarker assessment with validated functional, perceptual, and sport-specific performance outcomes.

### 4.3. Limitations and Strengths

Despite the methodological strengths of this review, several limitations should be acknowledged. First, substantial heterogeneity was observed in exercise protocols, participant characteristics, supplementation duration, dosage, and formulation type, limiting direct comparability across studies. Second, although athlete training status was classified using the McKay et al. framework, most trials were conducted in recreationally active or amateur individuals, with limited evidence in highly trained or professional athletes. Third, dietary intake and training-load monitoring were insufficiently controlled in most studies, increasing the risk of residual confounding. Fourth, verification of curcumin exposure and supplement composition was limited, as few trials measured circulating curcumin or independently confirmed supplement content. Fifth, many studies had small sample sizes, reducing statistical power, particularly in trained athletic populations. Sixth, the RoB 2 assessment reported in Supplementary Figures S1–S4 identified methodological or reporting-related concerns in several trials, particularly regarding incomplete reporting of randomization procedures, missing outcome data, selective reporting, and potential period or carryover effects in crossover designs. Consistently, the GRADE assessment indicated low or very low certainty of evidence across outcome domains, mainly because of risk-of-bias concerns, inconsistency, indirectness for some applied outcomes, and imprecision. These issues may reduce confidence in the direction and consistency of the findings. Finally, publication bias cannot be excluded, as most published trials reported at least one positive finding.

These limitations highlight the need for adequately powered trials with rigorous dietary and training-load control, standardized verification of supplement content and plasma curcumin levels, and broader representation of athlete populations.

This review also has several strengths. It focused specifically on exercise- and sport-related contexts in healthy active individuals and athletes, excluding artificial muscle-damage models with limited ecological validity. It also stratified findings by athlete training status, systematically evaluated dietary control and training-load monitoring, and applied strict inclusion criteria limited to randomized placebo-controlled trials. Finally, by integrating performance, muscle damage, inflammation, oxidative stress, and subjective recovery/fatigue-related outcomes, this review provides a comprehensive and sport-relevant synthesis of curcumin supplementation and exercise recovery.

### 4.4. Future Directions

Future research should prioritize well-controlled trials in highly trained and elite athletes, using sport-specific performance outcomes and recovery markers that reflect real training environments. Trials should also incorporate rigorous dietary standardization, objective training-load quantification, independent compositional analyses of supplements, and plasma assessments of curcumin and its metabolites. In addition, direct comparisons of supplementation timing, acute versus chronic regimens, and unformulated versus bioavailability-enhanced preparations are needed to determine whether specific dosing strategies or formulations provide meaningful advantages. Larger sample sizes, longer follow-up periods, and outcomes extending beyond biochemical biomarkers to include functional measures, sport performance indicators, and athlete-reported recovery metrics will be essential to clarify whether curcumin improves recovery in a way that translates into enhanced readiness and athletic performance.

## 5. Conclusions

Curcumin supplementation appears to offer potential benefits for exercise-related recovery in healthy active individuals and athletes, particularly through improvements in selected oxidative stress markers and some functional or subjective recovery/fatigue-related outcomes. However, these effects were not consistent across studies and varied according to training status, supplementation regimen, formulation type, and the degree of dietary and training-load control. The current evidence base is dominated by recreational and amateur populations, with limited data in highly trained or professional athletes, and few trials verified curcumin exposure or supplement composition analytically. Taken together, the findings suggest that curcumin may serve as a useful adjunct within recovery strategies, but current evidence is not yet sufficient to support broad practical recommendations for athletes. From an applied sport perspective, the most relevant evidence comes from a small subset of athlete-based match-play, competition-like, race-based, or field-based studies, suggesting potential benefits for selected recovery-related outcomes but highlighting the need for more ecologically valid trials in trained and high-performance athletes. Well-designed, adequately powered trials with rigorous methodological control are needed to clarify its role in supporting exercise recovery and performance.

## Figures and Tables

**Table 1:** PICOS criteria for study inclusion.

Element	Eligibility Criterion
Population	Healthy humans, including physically active individuals, recreationally active participants, amateur athletes, trained/developmental athletes, and professional/sub-elite athletes.
Intervention	Oral curcumin, curcuminoids, curcuma-derived preparations with a specified curcumin dose, or curcumin combined with bioavailability enhancers such as piperine.
Comparator	Placebo or control condition.
Outcomes	At least one exercise-related outcome: muscle damage, inflammation, oxidative stress, subjective recovery/soreness, fatigue, or physical performance.
Study design	Randomized double-blind placebo-controlled trials, including parallel and crossover designs.

**Table 2:** Methodological quality assessment of the included studies using the PEDro scale.

Author/s, Year	ELIG	RA	AC	BS	SB	TB	AB	FU	ITT	BG	PE	Total
Abbott et al. [39], 2023	1	1	1	0	1	1	0	1	1	1	1	8
Bańkowski et al. [40], 2025	1	1	0	0	1	1	0	0	0	1	1	5
Faria et al. [41], 2020	1	1	1	1	1	1	1	0	0	1	1	8
Ghojazadeh et al. [42], 2022	1	1	0	1	1	1	1	1	1	1	1	9
Juniarsyah et al. [43], 2024	1	1	0	1	1	1	0	0	0	1	1	6
Kisiolek et al. [44], 2022	1	1	0	0	1	1	0	1	1	1	1	7
Li et al. [45], 2025	1	1	1	1	1	1	0	1	0	1	1	8
Mallard et al. [46], 2021	1	1	0	1	1	1	0	1	0	1	1	7
McAllister et al. [47], 2020	1	1	0	0	1	1	0	0	0	1	1	5
Salehi et al. [48], 2021	1	1	0	0	1	1	0	0	0	1	1	5
Nakhostin Roohi et al. [49], 2016	1	1	0	1	1	1	0	1	0	1	1	7
Rosidi et al. [50], 2013	1	1	0	1	1	1	0	1	1	1	1	8
Sciberras et al. [51], 2015	1	1	0	0	1	1	0	1	0	1	1	6
Tanabe et al. [52], 2024	1	1	1	1	1	1	0	0	0	1	1	7
Takahashi et al. [53], 2014	1	1	0	1	1	1	0	0	0	1	1	6

Abbreviations: AB, assessor blinding; AC, allocation concealment; BG, between-group comparison; BS, baseline similarity; ELIG, eligibility criteria; FU, follow-up greater than 85%; ITT, intention-to-treat analysis; PEDro, Physiotherapy Evidence Database; PE, point estimates and variability; RA, random allocation; SB, subject blinding; TB, therapist/personnel blinding.

**Table 3:** Contextual, methodological, and real-world sport applicability characteristics of the included studies.

Study	Population/Training Status	Methodological Control	Curcumin Exposure or Supplement Verification	Real-World Sport Applicability
Abbott et al. [39], 2023	11 professional male soccer players; highly trained/professional athletes	Diet recorded; match demands monitored with GPS-derived external load	NR	High. Professional soccer match play with GPS-derived load and dietary intake recorded.
Bańkowski et al. [40], 2025	30 amateur long-distance runners; amateur/developmental athletes	Diet not clearly controlled; training period described, but objective load control limited	NR	Moderate. Amateur endurance runners during a preparatory phase, but outcomes assessed using a laboratory graded exercise test.
Faria et al. [41], 2020	28 amateur/recreational male runners; amateur/developmental athletes	Dietary intake/pre-race nutrition controlled or recorded; training volume recorded	Plasma curcumin or curcuminoids assessed	High. Real-world half-marathon race context with dietary intake and training volume recorded.
Ghojazadeh et al. [42], 2022	18 male taekwondo athletes; highly trained/professional athletes	Diet not clearly reported; competition protocol standardized	NR	High. Taekwondo athletes exposed to successive competition-like bouts, although simulated rather than official competition.
Juniarsyah et al. [43], 2024	16 amateur futsal players; amateur/developmental athletes	Diet not clearly controlled; training/match schedule described, objective load control limited	NR	High. Amateur futsal players exposed to repeated match-play demands.
Kisiolek et al. [44], 2022	36 physically active adults; recreationally active	Diet partially recorded; supervised HIIT, habitual activity not fully controlled	NR	Moderate. Structured high-intensity interval training and cycling time trial, but not a sport-specific athlete cohort.
Li et al. [45], 2025	71 healthy untrained adults; non-athlete population	Diet not clearly controlled; no structured training-load control	NR	Low. Healthy untrained adults and laboratory exercise test protocol.
Mallard et al. [46], 2021	27 strength-trained/recreationally active men	Diet around testing requested/partially controlled; standardized resistance protocol	NR	Low/indirect. Strength-trained men and laboratory-based resistance exercise to exhaustion.
McAllister et al. [47], 2020	14 physically active/trained men	Diet not clearly controlled; standardized laboratory challenge	NR	Low/indirect. Laboratory dual-stress model with limited direct transferability to sport recovery.
Salehi et al. [48], 2021	65 healthy women with moderate physical activity	Dietary intake assessed; physical activity habitual rather than standardized	NR	Low/indirect. Healthy women with moderate physical activity and no standardized sport-specific training load.
Nakhostin Roohi et al. [49], 2016	20 active healthy males; recreationally active	Diet not clearly controlled; running trial standardized	NR	Moderate. Field-relevant 14 km running trial, but participants were active healthy males rather than clearly defined athletes.
Rosidi et al. [50], 2013	35 male football athletes aged 14–18 years; amateur/developmental athletes	Food intake collected before/during treatment; physical activity level reported	NR	High. Football athletes assessed using a field-based 5000 m running test.
Sciberras et al. [51], 2015	11 recreationally active males	Standardized pre-trial diet; standardized exercise protocol	Plasma curcumin assessed	Moderate. Controlled endurance cycling model in recreationally active males, with standardized exercise protocol and plasma curcumin assessment.
Tanabe et al. [52], 2024	15 collegiate soccer players; amateur/developmental athletes	Daily energy/nutrient intake assessed; GPS and heart-rate match-load monitoring	NR	High. Collegiate soccer match play with objective match-load monitoring.
Takahashi et al. [53], 2014	10 healthy/recreationally active men	Same diet on trial day; standardized treadmill protocol	Plasma curcumin assessed	Low/indirect. Healthy/recreationally active men and treadmill-based laboratory oxidative-stress model.

Note. Methodological control summarizes the available information on dietary control, exercise or training-load monitoring, and/or standardization of the exercise or sport protocol as reported in each study. Curcumin exposure or supplement verification refers to the assessment of circulating curcumin/curcuminoids and/or verification of supplement composition when reported. Real-world sport applicability was classified as high, moderate, or low/indirect according to the predefined criteria described in Section 2.3. Abbreviations: GPS, Global Positioning System; HIIT, high-intensity interval training; NR, not reported.

**Table 4:** Main curcumin formulation technologies relevant to the review.

Technology/Formulation	Composition	Mechanism of Bioavailability Enhancement	Reported Bioavailability Increase vs. Unformulated Curcumin	Sample Hydrolyzed
Curcumin + piperine	Curcumin co-administered with piperine	Inhibition of hepatic and intestinal glucuronidation	20-fold in classic pharmacokinetic work	No [10]
Nanoparticle colloidal dispersion	Curcumin with other curcuminoids, glycerin, water, and gum ghatti	Improved solubility, stability, and reduced particle size	27-fold	Yes [55]
Micellar curcumin	Curcumin powder dispersed in micellar carrier	Improved aqueous solubility	185-fold	Yes [56]
Native turmeric matrix from fresh rhizome	Curcumin in native amorphous complexed form	Enhanced aqueous dispersibility and protection from metabolic conjugation	40-fold	No [57]
Solid lipid curcumin particle	Curcumin/turmeric extract with lipid and phospholipid components	Improved lipid-based dispersion, stability, and absorption	100-fold	No [58]
Amorphous curcumin	Amorphous curcumin with diluents and lubricants	Enhanced solubility	85-fold	Yes [59]
Natural turmeric matrix	Curcuminoids combined with turmeric-derived carbohydrates, proteins, oils, and dietary fiber	Improved stability, controlled release, and aqueous dispersibility	5.5-fold	No [60]
Phytosome	Curcuminoid-lecithin complex	Improved stability and lipid-compatible delivery	18-fold	Yes [61]
Dispersion delivery system	Curcuminoids dispersed using wetting/dispersion technology	Increased wettability and dispersibility, preventing agglomeration	2.2-fold	Yes [62]

**Table 5:** Direction of findings according to supplementation timing.

Supplementation Pattern	No. of Studies/Comparisons	Performance Outcomes	Muscle Damage	Inflammation	Oxidative Stress	Subjective Recovery/Fatigue-Related Outcomes
**Acute/peri-exercise**	7	2/3	1/3	1/4	2/3	3/6
Acute pre-exercise	3	-	-	0/1	1/2	1/3
Acute post-exercise	1	1/1	-	-	-	1/1
Acute pre + post-exercise	4	1/2	1/3	1/3	2/2	1/3
**Chronic/short-to-long-term**	8	2/5	3/3	2/4	4/4	0/1
Total	15	4/8	4/6	3/8	6/7	3/7

**Note.** Values are expressed as the number of studies or comparisons reporting a favorable effect divided by the number of studies or comparisons assessing the corresponding outcome domain. The overall acute/peri-exercise row and the total row were calculated at the study level to avoid double-counting individual trials, whereas timing-specific subrows were calculated at the comparison level when one study contributed more than one supplementation-timing condition. Acute/peri-exercise protocols included Abbott et al. [39], Ghojazadeh et al. [42], Mallard et al. [46], McAllister et al. [47], Sciberras et al. [51], Takahashi et al. [53], and Tanabe et al. [52]. Chronic/short-to-long-term protocols included Bańkowski et al. [40], Faria et al. [41], Juniarsyah et al. [43], Kisiolek et al. [44], Li et al. [45], Nakhostin Roohi et al. [49], Rosidi et al. [50], and Salehi et al. [48]. A favorable effect was defined as a statistically significant between-group difference or group-by-time interaction in favor of curcumin compared with placebo or control for at least one relevant outcome within the corresponding domain. Within-group changes without between-group evidence were not considered favorable. “-” indicates that the outcome domain was not assessed.

**Table 6:** Summary of randomized double-blind placebo-controlled trials evaluating the effects of curcumin supplementation on exercise recovery and related biomarkers.

Study	Supplementation Protocol	Exercise Context	Outcome Domains Assessed	Overall Direction of Findings	Main Interpretation
Abbott et al. [39], 2023	500 mg curcumin immediately, 12 h, and 36 h after match play	Professional 90 min soccer match	Performance/function; subjective recovery	Favorable	Faster recovery of CMJ, RSI, and DOMS; no biomarker assessment.
Bańkowski et al. [40], 2025	2 g/day turmeric extract + piperine for 6 weeks	Preparatory endurance-running phase plus graded exercise test	Inflammation; blood count; BDNF	Mostly neutral	No clear effect on inflammatory markers or blood count; BDNF increased numerically.
Faria et al. [41], 2020	1.5 g/day Curcuma longa extract for 4 weeks plus peri-race dosing	Half-marathon race	Inflammation; muscle damage; race performance	Partially favorable	Higher IL-10 and lower myoglobin; no clear effects on CK, AST, ALT, LDH, IL-6, or race time.
Ghojazadeh et al. [42], 2022	4 g/day curcumin from 3 days before to 2 days after competition	Three successive simulated taekwondo competitions	Muscle damage; inflammation; oxidative stress	Favorable for selected domains	Attenuated CK, LDH, and MDA and increased TAC; no clear IL-6 effect.
Juniarsyah et al. [43], 2024	200 mg/day curcumin + 10 mg/day piperine for 14 days	Training week plus two consecutive futsal matches	Muscle damage; performance	Favorable	Lower CK, AST, and ALT at selected post-match time points. Improved CMJ and 20-m sprint performance.
Kisiolek et al. [44], 2022	1 g/day optimized solid lipid curcumin for 2 weeks	Supervised HIIT plus 16.1 km cycling time trial	Performance; lactate; wellbeing; inflammation	Mostly neutral/mixed	No clear between-group effects; no evidence of impaired performance adaptation.
Li et al. [45], 2025	120 mg/day amorphous curcumin for 8 weeks	Laboratory cardiorespiratory exercise testing	Oxidative stress; performance	Favorable for selected oxidative markers	Lower TOS and OSI; no clear MDA or 8-OHdG effect; no clear time-to-exhaustion effect.
Mallard et al. [46], 2021	500 mg dispersion-delivery curcumin before and after exercise, plus 24 and 48 h doses	Resistance exercise to exhaustion	Soreness; lactate; muscle damage; inflammation	Partially favorable	Lower DOMS, thigh circumference, and lactate; biomarker effects mixed, with no clear CK, LDH, myoglobin, hs-CRP, or TNF-alpha effect.
McAllister et al. [47], 2020	1.5 g/day native amorphous curcumin for 3 days plus pre-test dose	Cycling plus mental stress challenge	Oxidative stress; exertion responses	Neutral	No clear treatment effect on oxidative stress markers, RPE, or heart rate.
Salehi et al. [48], 2021	500 mg/day curcumin for 8 weeks	Habitual moderate physical activity	Inflammation; oxidative stress; LDH; VO_2_max	Favorable for selected outcomes	Lower CRP, LDH, and MDA and higher VO_2_max; no clear TAC effect.
Nakhostin Roohi et al. [49], 2016	90 mg/day curcumin for 7 days	14 km running trial	Oxidative stress	Favorable	Improved TAC and GSH-related responses and reduced MDA/TBARS responses.
Rosidi et al. [50], 2013	Temulawak extract providing 250–750 mg/day curcumin for 21 days	5000 m running test in football athletes	Oxidative stress	Favorable	MDA increased in placebo and decreased in curcumin groups; no clear between-dose differences.
Sciberras et al. [51], 2015	500 mg curcumin phytosome for 3 days plus pre-exercise dose	2 h endurance cycling	Inflammation; stress markers; subjective recovery	Mostly neutral/partially favorable	No clear inflammatory effects; IL-6 tended lower and DALDA responses favored curcumin.
Tanabe et al. [52], 2024	Nanoparticle colloidal dispersion: five 90 mg doses over 48 h	Controlled collegiate soccer match	Soreness; performance; CRP; CK; urinary titin	Neutral	No clear between-condition differences for soreness, jump outcomes, CRP, CK, or urinary titin.
Takahashi et al. [53], 2014	90 mg nanoparticle colloidal dispersion pre-exercise or pre + post-exercise	60 min treadmill exercise at 65% VO_2_max	Oxidative stress; antioxidant response; exertion responses	Favorable for selected oxidative markers	Exercise-related d-ROMs/TRX-1 increases occurred mainly in placebo; BAP/GSH responses favored curcumin; other markers neutral.

Note. All included studies were randomized double-blind placebo-controlled trials. Abbreviations: 8-OHdG, 8-hydroxy-2-deoxyguanosine; ALT, alanine aminotransferase; AST, aspartate aminotransferase; BAP, biological antioxidant potential; BDNF, brain-derived neurotrophic factor; CK, creatine kinase; CMJ, countermovement jump; CRP, C-reactive protein; DALDA, Daily Analysis of Life Demands for Athletes; DOMS, delayed-onset muscle soreness; GSH, glutathione; HIIT, high-intensity interval training; hs-CRP, high-sensitivity C-reactive protein; IL, interleukin; LDH, lactate dehydrogenase; MDA, malondialdehyde; OSI, oxidative stress index; RPE, rating of perceived exertion; RSI, reactive strength index; TAC, total antioxidant capacity; TBARS, thiobarbituric acid-reactive substances; TNF-alpha, tumor necrosis factor-alpha; TOS, total oxidant status; TRX-1, thioredoxin-1. The direction of findings is descriptive and reflects at least one favorable finding within the corresponding outcome domain; it should not be interpreted as indicating consistent improvement across all biomarkers, time points, or outcomes.

**Table 7:** Direction of findings according to participant training status.

Training Status	No. of Studies	Performance Outcomes	Muscle Damage	Inflammation	Oxidative Stress	Subjective Recovery/Fatigue-Related Outcomes
Healthy untrained or moderately active non-athletes (tier 0)	1	0/1	-	-	1/1	-
Recreationally active participants (tier 1)	7	2/3	1/2	2/4	3/4	2/5
Amateur/developmental athletes (tier 2)	5	1/3	2/3	1/3	1/1	0/1
Highly trained/professional athletes (tier 3)	2	1/1	1/1	0/1	1/1	1/1
Total	15	4/8	4/6	3/8	6/7	3/7

Note. Values indicate the number of studies reporting a favorable effect divided by the number of studies assessing each outcome domain. All values in this table were calculated at the study level, so each trial was counted only once within its corresponding training-status category. Healthy untrained/non-athlete adults included Li et al. [45]. Recreationally active studies included Mallard et al. [46], Sciberras et al. [51], Takahashi et al. [53], Nakhostin Roohi et al. [49], McAllister et al. [47], Kisiolek et al. [44], and Salehi et al. [48]. Amateur/developmental athlete studies included Juniarsyah et al. [43], Faria et al. [41], Bańkowski et al. [40], Tanabe et al. [52], and Rosidi et al. [50]. Highly trained/professional evidence was represented by Abbott et al. [39] and Ghojazadeh et al. [42]. A favorable effect was defined as a statistically significant between-group difference or group-by-time interaction in favor of curcumin compared with placebo or control for at least one relevant outcome within the corresponding domain. Within-group changes without between-group evidence were not considered favorable. “-” indicates that the outcome domain was not assessed.

## Data Availability

No new datasets were generated or analyzed in this study. All data supporting the findings of this systematic review are available in the included articles and Supplementary Materials.

## Supplementary Materials

The following supporting information can be downloaded at: https://www.mdpi.com/article/10.3390/nu18121992/s1, Table S1: Complete electronic search strategies; Table S2: PRISMA 2020 checklist; Table S3: Studies excluded after full-text assessment and reasons for exclusion; Table S4: Commercial curcumin products, formulation categories, bioavailability-enhancement strategies, and exposure/verification data in the included studies; Table S5: GRADE certainty assessment by outcome domain; Table S6. Real-world applicability classification of the included studies. Figure S1: RoB 2 assessment for crossover trials, intention-to-treat effect; Figure S2: RoB 2 assessment for parallel-group trials, intention-to-treat effect; Figure S3: RoB 2 assessment for crossover trials, per-protocol effect; Figure S4: RoB 2 assessment for parallel-group trials, per-protocol effect.

## Abbreviations

The following abbreviations are used in this manuscript:

PRISMA	Preferred Reporting Items for Systematic Reviews and Meta-Analyses
PICOS	Population, Intervention, Comparator, Outcomes, and Study design
PEDro	Physiotherapy Evidence Database
ROS	Reactive oxygen species
CK	Creatine kinase
CRP	C-reactive protein
IL-6	Interleukin-6
VO_2_max	Maximal oxygen uptake
