# Supplementary material for: Effects of Curcumin Supplementation on Exercise Recovery, Oxidative Stress, Inflammation, Muscle Damage, and Performance in Exercise and Sport Contexts: A Systematic Review
Source: Nutrients. 2026 Jun 19;18(12):1992. doi: 10.3390/nu18121992 (PMC13304679; doi:10.3390/nu18121992)
Supplement: Supplementary file 1 [file nutrients-18-01992-s001.zip › Sup table S6.docx]

**Supplementary Table S6**. Real-world applicability classification of the included studies.

| Study | Real-world applicability | Rationale |
| --- | --- | --- |
| Abbott et al. (2023) | High | Conducted in male professional soccer players after a soccer match; outcomes included delayed onset muscle soreness and muscle function, directly relevant to applied recovery. |
| Bańkowski et al. (2025) | Moderate | Conducted in amateur long-distance runners during the preparatory phase; sport-specific population, but the main assessment relied on a graded treadmill exercise test and inflammatory/hematological outcomes. |
| Faria et al. (2020) | High | Conducted in recreational male runners after a half-marathon race; the protocol reflected a real endurance competition and assessed inflammation and muscle damage markers. |
| Ghojazadeh et al. (2022) | High | Conducted in trained taekwondo athletes after successive simulated competitions; sport-specific high-intensity context with recovery-related biomarkers. |
| Juniarsyah et al. (2024) | High | Conducted in futsal players after two consecutive futsal matches; protocol closely reflected congested match-play demands and assessed recovery-related outcomes. |
| Kisiolek et al. (2021) | Moderate | Conducted in the context of high-intensity interval training; relevant to exercise training adaptations, but less directly linked to competition or acute sport recovery. |
| Li et al. (2025) | Low/indirect | Conducted in healthy adults using a laboratory-based exercise protocol focused mainly on oxidative stress biomarkers; limited direct transferability to athletes or sport competition. |
| Mallard et al. (2021) | Low/indirect | Conducted in recreationally trained males using lower-limb resistance exercise to exhaustion; relevant to exercise recovery, but not sport-specific or competition-based. |
| McAllister et al. (2020) | Low/indirect | Conducted in trained men using a controlled dual-stress laboratory challenge; outcomes were mainly oxidative stress-related, limiting direct applied sport translation. |
| Nakhostin Roohi et al. (2016) | Moderate | Conducted in active healthy males after a 14-km running protocol; relevant to exercise-induced oxidative stress, although not clearly competition-based. |
| Rosidi et al. (2013) | High | Conducted in young football athletes and focused on oxidative stress markers; sport-specific sample, but outcomes were mainly biochemical and practical recovery or performance outcomes were limited. |
| Salehi et al. (2021) | Low | Conducted in healthy females with moderate physical activity; outcomes included inflammatory, oxidative stress, muscle damage, and VO₂max-related variables, but the population and setting had limited direct applicability to sport recovery. |
| Sciberras et al. (2015) | Moderate | Conducted in recreational athletes during endurance cycling; the protocol was exercise-related and recovery-relevant, but laboratory-based and with limited sport-specific performance outcomes. |
| Takahashi et al. (2014) | Low/indirect | Acute laboratory-based endurance exercise study in healthy male participants, focused mainly on oxidative stress biomarkers. |
| Tanabe et al. (2024) | High | Conducted in collegiate soccer players after soccer matches, with assessment of muscle soreness, jump performance, inflammatory markers, and muscle damage markers; highly relevant to applied team-sport recovery. |
| Overall interpretation | Moderate to low overall applicability | Although several studies were conducted in sport-specific contexts, most evidence came from recreational, amateur, or controlled laboratory settings, and many outcomes were biochemical rather than direct measures of sport performance or recovery. |
