# Supplementary material for: Effects of Curcumin Supplementation on Exercise Recovery, Oxidative Stress, Inflammation, Muscle Damage, and Performance in Exercise and Sport Contexts: A Systematic Review
Source: Nutrients. 2026 Jun 19;18(12):1992. doi: 10.3390/nu18121992 (PMC13304679; doi:10.3390/nu18121992)
Supplement: Supplementary file 1 [file nutrients-18-01992-s001.zip › Table S3. Excluded studies curcumin.docx]

**Table S3.** Studies excluded after full-text assessment and reasons for exclusion

| Study | Citation | Reason for exclusion |
| --- | --- | --- |
| Clayton et al. (2023) | Clayton D, Burbeary R, Hennis P, et al. Turmeric supplementation improves markers of recovery in elite male footballers: a pilot study. Frontiers in Nutrition. 2023;10:1175622. doi:10.3389/fnut.2023.1175622 | Non-randomized and non-placebo-controlled pilot study. Although conducted in professional footballers and relevant to applied sport recovery, the absence of random allocation, placebo control, and blinding precluded inclusion in the main synthesis of randomized placebo-controlled trials. |
| Choi et al. (2023) | Choi Y, Ra SG, Nishijima T, Maeda S. Effect of curcumin supplementation on inflammatory status and muscle damage in competitive female soccer players: a placebo-controlled, single-blind, nonrandomized, crossover pilot study. Physical Activity and Nutrition. 2023;27(2):34-38. doi:10.20463/pan.2023.0016 | Non-randomized, single-blind crossover pilot study with a very small sample. The study was relevant to competitive soccer, but did not meet the randomized trial criterion. |
| Goh et al. (2020) | Goh J, Menke W, Herrick LP, et al. Examination of curcumin and fenugreek soluble fiber supplementation on submaximal and maximal aerobic performance indices. Journal of Functional Morphology and Kinesiology. 2020;5(2):34. doi:10.3390/jfmk5020034 | Combined curcumin-fenugreek soluble fiber intervention. Because fenugreek/galactomannan may have independent physiological effects and the fenugreek-only group also showed changes in ventilatory threshold, the effect could not be attributed clearly to curcumin alone. |
| Herrick et al. (2020) | Herrick LP, Goh J, Menke W, et al. Effects of curcumin and fenugreek soluble fiber on the physical working capacity at the fatigue threshold, peak oxygen consumption, and time to exhaustion. Journal of Strength and Conditioning Research. 2020;34(12):3346-3355. doi:10.1519/JSC.0000000000003852 | Combined curcumin-fenugreek soluble fiber intervention. The independent contribution of curcumin could not be isolated, and the outcomes were mainly aerobic performance/fatigue-threshold indices rather than exercise-recovery biomarkers attributable to curcumin alone. |
| Varma et al. (2021) | Varma K, Amalraj A, Divya C, Gopi S. The efficacy of the novel bioavailable curcumin (Cureit) in the management of sarcopenia in healthy elderly subjects: a randomized, placebo-controlled, double-blind clinical study. Journal of Medicinal Food. 2021;24(1):40-49. doi:10.1089/jmf.2020.4778 | Population and outcomes focused on sarcopenia management or non-exercise-related muscle function in older adults. The study did not evaluate exercise-related recovery, training, competition, or post-exercise responses. |
| Juniarsyah et al. (2025) | Juniarsyah AD, Apriantono T, Kurniati NF, et al. Recovery effect of turmeric (Curcuma longa L.) and black pepper (Piper nigrum) in combination on futsal players. Retos. 2025;64:913-924. doi:10.47197/retos.v64.108221 | Companion or duplicate report from the same or highly overlapping futsal supplementation protocol and sample as Juniarsyah et al. (2024). It was not counted as an independent study to avoid double-counting participants. |
| Tanabe et al. (2019a) | Tanabe Y, Chino K, Ohnishi T, et al. Effects of oral curcumin ingested before or after eccentric exercise on markers of muscle damage and inflammation. Scandinavian Journal of Medicine & Science in Sports. 2019;29(4):524-534. doi:10.1111/sms.13373 | Artificial muscle-damage protocol: isolated maximal eccentric elbow-flexor exercise designed primarily to induce substantial muscle damage rather than reflect habitual training, competition, or sport-recovery practice. Although informative for supplementation timing, the study did not meet the applied exercise/sport-context eligibility criterion of the present review. |
| Tanabe et al. (2019b) | Tanabe Y, Chino K, Sagayama H, et al. Effective timing of curcumin ingestion to attenuate eccentric exercise-induced muscle soreness in men. Journal of Nutritional Science and Vitaminology. 2019;65(1):82-89. doi:10.3177/jnsv.65.82 | Artificial muscle-damage protocol: isolated eccentric elbow-flexor exercise designed primarily to induce substantial muscle damage rather than reflect habitual training, competition, or sport-recovery practice. The study was therefore excluded from the main synthesis, although it was considered only as contextual evidence regarding supplementation timing. |

Abbreviations: PICOS, population, intervention, comparator, outcomes, and study design.
