# Supplementary material for: Effects of Curcumin Supplementation on Exercise Recovery, Oxidative Stress, Inflammation, Muscle Damage, and Performance in Exercise and Sport Contexts: A Systematic Review
Source: Nutrients. 2026 Jun 19;18(12):1992. doi: 10.3390/nu18121992 (PMC13304679; doi:10.3390/nu18121992)
Supplement: Supplementary file 1 [file nutrients-18-01992-s001.zip › Table S4.docx]

**Table S4.** Commercial curcumin products, formulation categories, bioavailability-enhancement strategies, and exposure/verification data in the included studies.

| Commercial formulation | Formulation category / technology | Proposed bioavailability-enhancement strategy | Studies using this formulation | Exposure or verification data reported in the included trials |
| --- | --- | --- | --- | --- |
| Curcumin + piperine | Curcumin combined with piperine | Piperine may enhance curcumin bioavailability by inhibiting intestinal and hepatic glucuronidation | Juniarsyah et al., 2024; Bańkowski et al., 2025 | Circulating curcumin exposure and supplement composition were not reported in the included trials |
| Theracurmin® | Nanoparticle colloidal dispersion | Reduced particle size and improved aqueous dispersion of curcumin | Tanabe et al., 2024; Takahashi et al., 2014 | Plasma curcumin was assessed in Takahashi et al.; circulating exposure was not consistently assessed across studies |
| Theracurmin Super® | Enhanced-bioavailability curcumin preparation / amorphous curcumin-based formulation | Improved solubility and systemic exposure compared with unformulated curcumin | Li et al., 2025 | Circulating curcumin exposure and supplement composition were not reported in the included trial |
| NovaSOL® | Micellar curcumin | Improved aqueous solubility through micellar delivery | Abbott et al., 2023 | Circulating curcumin exposure and supplement composition were not reported in the included trial |
| Longvida® | Solid lipid curcumin particle | Lipid-based delivery intended to improve stability, absorption, and systemic exposure | Kisiolek et al., 2022; McAllister et al., 2020 | Circulating curcumin exposure and supplement composition were not reported in the included trials |
| Meriva® | Curcumin phytosome | Curcuminoid–phospholipid complex designed to improve lipid-compatible delivery and absorption | Sciberras et al., 2015 | Plasma curcumin was assessed in the included trial |
| HydroCurc® | Dispersion delivery system | Improved wettability and dispersibility, reducing agglomeration and supporting absorption | Mallard et al., 2021 | Circulating curcumin exposure and supplement composition were not reported in the included trial |
| Unbranded standardized curcumin or Curcuma-derived extract | Standard curcumin/turmeric-derived preparation | No specific commercial bioavailability-enhancement technology reported | Faria et al., 2020; Ghojazadeh et al., 2022; Nakhostin Roohi et al., 2016; Rosidi et al., 2013; Salehi et al., 2021 | Plasma curcumin or curcuminoids were assessed in Faria et al.; circulating exposure was not reported in the remaining trials |
