# Supplementary material for: Effects of Curcumin Supplementation on Exercise Recovery, Oxidative Stress, Inflammation, Muscle Damage, and Performance in Exercise and Sport Contexts: A Systematic Review
Source: Nutrients. 2026 Jun 19;18(12):1992. doi: 10.3390/nu18121992 (PMC13304679; doi:10.3390/nu18121992)
Supplement: Supplementary file 1 [file nutrients-18-01992-s001.zip › Table S5.docx]

**Supplementary Table S5.** GRADE certainty assessment by outcome domain

**Question:** Should oral curcumin supplementation, compared with placebo/control, be used to improve exercise recovery-related outcomes in healthy active individuals and athletes?

| **Certainty assessment** | | | | | | | **№ of patients** | | **Effect** | | **Certainty** | **Importance** |
| --- | --- | --- | --- | --- | --- | --- | --- | --- | --- | --- | --- | --- |
| **№ of studies** | **Study design** | **Risk of bias** | **Inconsistency** | **Indirectness** | **Imprecision** | **Other considerations** | **oral curcumin supplementation** | **control** | **Relative (95% CI)** | **Absolute (95% CI)** |  |  |
| **Oxidative stress (assessed with: MDA, TAC, TOS, OSI, GSH-related markers, SOD, d-ROMs, BAP, TBARS, 8-OHdG, Trx-1)** | | | | | | | | | | | | |
| 7 | randomised trials | serious^a^ | not serious | not serious | serious^b^ | none | Seven trials involving 233 participants assessed oxidative stress-related outcomes. Six of seven studies reported at least one favorable effect of curcumin on oxidative stress or antioxidant-response markers. Findings were generally more consistent than for other outcome domains, particularly for markers such as malondialdehyde, total antioxidant capacity, total oxidant status, oxidative stress index, glutathione-related markers, derivatives of reactive oxygen metabolites, and biological antioxidant potential. However, biomarkers, timing of assessment, exercise protocols, participant characteristics, and curcumin formulations differed substantially across studies. | | | | ⨁⨁◯◯ Low^a,b^ | IMPORTANT |
| **Muscle damage (assessed with: CK, LDH, myoglobin, AST, ALT, urinary N-terminal fragment of titin)** | | | | | | | | | | | | |
| 6 | randomised trials | serious^c^ | serious^d^ | not serious | serious^e^ | none | Six trials involving 169 participants assessed muscle-damage-related outcomes. Four of six studies reported at least one favorable effect of curcumin on markers of muscle damage, whereas others found no clear between-group differences. Favorable findings were mainly observed for selected markers such as creatine kinase, lactate dehydrogenase, myoglobin, aspartate aminotransferase, or alanine aminotransferase, but results varied according to the biomarker assessed, timing of blood sampling, exercise protocol, training status, and formulation. | | | | ⨁◯◯◯ Very low^c,d,e^ | CRITICAL |
| **Inflammation (assessed with: CRP, hs-CRP, IL-6, IL-10, IL-1β, IL-1ra, TNF-α, blood count, neutrophils, white blood cells)** | | | | | | | | | | | | |
| 8 | randomised trials | serious^c^ | serious^f^ | not serious | serious^g^ | none | Eight trials involving 230 participants assessed inflammatory outcomes. Three of eight studies reported at least one favorable effect of curcumin on inflammatory markers, whereas most studies showed no clear between-group differences for C-reactive protein, high-sensitivity C-reactive protein, interleukin-6, interleukin-1β, interleukin-10, tumor necrosis factor-alpha, or related immune-cell markers. Interpretation was limited by differences in exercise stress, sampling time points, baseline training status, inflammatory biomarkers assessed, and curcumin formulations. | | | | ⨁◯◯◯ Very low^c,f,g^ | IMPORTANT |
| **Subjective recovery, soreness, and fatigue (assessed with: DOMS, muscle soreness, perceived recovery, subjective wellbeing, fatigue, RPE, DALDA questionnaire)** | | | | | | | | | | | | |
| 7 | randomised trials | serious^c^ | serious^h^ | serious^i^ | serious^j^ | none | Seven trials involving 124 participants assessed subjective recovery, soreness, fatigue, or perceived exertion-related outcomes. Three of seven studies reported at least one favorable effect of curcumin, mainly for delayed onset muscle soreness, subjective wellbeing, or recovery-related perceptions. However, findings were mixed, and several studies reported no clear between-group differences. The evidence was limited by heterogeneity in subjective scales, timing of assessment, exercise context, participant training status, and small sample sizes. | | | | ⨁◯◯◯ Very low^c,h,i,j^ | IMPORTANT |
| **Physical or athletic performance (assessed with: CMJ, RSI, jump performance, sprint, time trial, race time, VO₂max, time to exhaustion, peak torque, power, neuromuscular function)** | | | | | | | | | | | | |
| 8 | randomised trials | serious^c^ | serious^k^ | serious^l^ | serious^m^ | none | Eight trials involving 288 participants assessed physical or athletic performance outcomes. Four of eight studies reported at least one favorable effect of curcumin on selected performance or functional outcomes, such as countermovement jump, reactive strength index, neuromuscular function, VO₂max, race-related performance, or recovery of performance after exercise. However, effects were inconsistent and outcomes were highly heterogeneous. Improvements in biomarker-based outcomes did not consistently translate into clear performance benefits. | | | | ⨁◯◯◯ Very low^c,k,l,m^ | CRITICAL |

**CI:** confidence interval

#### Explanations

a. Downgraded one level because several trials had some concerns or high risk of bias in the supplementary RoB 2 assessment, mainly due to incomplete reporting of randomisation procedures, selective reporting, missing outcome data, or potential period/carryover effects in crossover designs.

b. Downgraded one level because most trials had small sample sizes, no pooled effect estimate was available, and estimates were derived from heterogeneous biomarkers and time points.

c. Downgraded one level because several trials had some concerns or high risk of bias in the supplementary RoB 2 assessment.

d. Downgraded one level because findings were inconsistent across muscle damage markers, including creatine kinase, lactate dehydrogenase, myoglobin, aspartate aminotransferase, alanine aminotransferase, and urinary N-terminal fragment of titin.

e. Downgraded one level because the number of participants was small, no pooled estimate was available, and the results varied across biomarkers and time points.

f. Downgraded one level because findings were inconsistent across inflammatory markers, including C-reactive protein, high-sensitivity C-reactive protein, interleukin-6, interleukin-10, interleukin-1β, tumour necrosis factor-alpha, and related immune-cell markers.

g. Downgraded one level because most trials had small sample sizes, the number of studies per inflammatory marker was limited, and no pooled effect estimate was available.

h. Downgraded one level because findings varied across subjective outcomes, including delayed onset muscle soreness, perceived recovery, fatigue, perceived exertion, wellbeing, and recovery-related questionnaires.

i. Downgraded one level because subjective recovery outcomes were assessed using heterogeneous scales and in different exercise contexts, many of which were based on recreationally active or amateur participants rather than highly trained or professional athletes.

j. Downgraded one level because most trials had small sample sizes, no pooled effect estimate was available, and subjective outcomes were assessed at heterogeneous time points.

k. Downgraded one level because effects were inconsistent across heterogeneous performance outcomes, including countermovement jump, reactive strength index, sprint performance, time trial, race-related performance, VO₂max, time to exhaustion, peak torque, power, and neuromuscular function.

l. Downgraded one level because performance outcomes were highly heterogeneous and did not always reflect direct real-world sport performance, especially in highly trained or professional athletes.

m. Downgraded one level because most studies had small sample sizes, no pooled effect estimate was available, and confidence intervals or study-level estimates were insufficient to support precise conclusions.
