# Supplementary material for: Effects of Curcumin Supplementation on Exercise Recovery, Oxidative Stress, Inflammation, Muscle Damage, and Performance in Exercise and Sport Contexts: A Systematic Review
Source: Nutrients. 2026 Jun 19;18(12):1992. doi: 10.3390/nu18121992 (PMC13304679; doi:10.3390/nu18121992)
Supplement: Supplementary file 1 [file nutrients-18-01992-s001.zip › Table_S1_revised_search_strategy_specific.docx]

**Supplementary Table S1. Complete electronic search strategies**

| Database | Last search date | Complete search strategy | Records retrieved |
| --- | --- | --- | --- |
| PubMed | 12 June 2026 | (("Curcumin"[Mesh] OR curcumin*[Title/Abstract] OR curcuminoid*[Title/Abstract] OR turmeric[Title/Abstract] OR "Curcuma longa"[Title/Abstract]) AND ("Exercise"[Mesh] OR "Sports"[Mesh] OR "Athletes"[Mesh] OR exercis*[Title/Abstract] OR sport*[Title/Abstract] OR athlete*[Title/Abstract] OR "physical activity"[Title/Abstract] OR "physical activities"[Title/Abstract] OR "resistance training"[Title/Abstract] OR "strength training"[Title/Abstract] OR "endurance training"[Title/Abstract] OR "aerobic exercise"[Title/Abstract] OR "high-intensity exercise"[Title/Abstract] OR "high intensity interval training"[Title/Abstract] OR HIIT[Title/Abstract] OR running[Title/Abstract] OR runner*[Title/Abstract] OR marathon[Title/Abstract] OR cycling[Title/Abstract] OR soccer[Title/Abstract] OR football[Title/Abstract] OR futsal[Title/Abstract] OR competition[Title/Abstract] OR match[Title/Abstract]) AND (recover*[Title/Abstract] OR "exercise recovery"[Title/Abstract] OR "recovery intervention"[Title/Abstract] OR "muscle damage"[Title/Abstract] OR "exercise-induced muscle damage"[Title/Abstract] OR EIMD[Title/Abstract] OR DOMS[Title/Abstract] OR "delayed onset muscle soreness"[Title/Abstract] OR soreness[Title/Abstract] OR fatigue[Title/Abstract] OR "Oxidative Stress"[Mesh] OR "oxidative stress"[Title/Abstract] OR "Inflammation"[Mesh] OR inflamm*[Title/Abstract] OR cytokine*[Title/Abstract] OR "C-reactive protein"[Title/Abstract] OR CRP[Title/Abstract] OR "creatine kinase"[Title/Abstract] OR CK[Title/Abstract] OR "lactate dehydrogenase"[Title/Abstract] OR LDH[Title/Abstract] OR "physical performance"[Title/Abstract] OR "athletic performance"[Title/Abstract] OR "training adaptation"[Title/Abstract] OR "training adaptations"[Title/Abstract])) | 318 |
| Scopus | 12 June 2026 | TITLE-ABS-KEY (curcumin* OR curcuminoid* OR turmeric OR "Curcuma longa") AND TITLE-ABS-KEY (exercis* OR sport* OR athlete* OR "physical activity" OR "physical activities" OR "resistance training" OR "strength training" OR "endurance training" OR "aerobic exercise" OR "high-intensity exercise" OR "high intensity interval training" OR HIIT OR running OR runner* OR marathon OR cycling OR soccer OR football OR futsal OR competition OR match) AND TITLE-ABS-KEY (recover* OR "exercise recovery" OR "recovery intervention" OR "muscle damage" OR "exercise-induced muscle damage" OR EIMD OR DOMS OR "delayed onset muscle soreness" OR soreness OR fatigue OR "oxidative stress" OR inflamm* OR cytokine* OR "C-reactive protein" OR CRP OR "creatine kinase" OR CK OR "lactate dehydrogenase" OR LDH OR "physical performance" OR "athletic performance" OR "training adaptation" OR "training adaptations") | 930 |
| Web of Science | 12 June 2026 | ((TI=(curcumin* OR curcuminoid* OR turmeric OR "Curcuma longa") OR AB=(curcumin* OR curcuminoid* OR turmeric OR "Curcuma longa")) AND (TI=(exercis* OR sport* OR athlete* OR "physical activity" OR "physical activities" OR "resistance training" OR "strength training" OR "endurance training" OR "aerobic exercise" OR "high-intensity exercise" OR "high intensity interval training" OR HIIT OR running OR runner* OR marathon OR cycling OR soccer OR football OR futsal OR competition OR match) OR AB=(exercis* OR sport* OR athlete* OR "physical activity" OR "physical activities" OR "resistance training" OR "strength training" OR "endurance training" OR "aerobic exercise" OR "high-intensity exercise" OR "high intensity interval training" OR HIIT OR running OR runner* OR marathon OR cycling OR soccer OR football OR futsal OR competition OR match)) AND (TI=(recover* OR "exercise recovery" OR "recovery intervention" OR "muscle damage" OR "exercise-induced muscle damage" OR EIMD OR DOMS OR "delayed onset muscle soreness" OR soreness OR fatigue OR "oxidative stress" OR inflamm* OR cytokine* OR "C-reactive protein" OR CRP OR "creatine kinase" OR CK OR "lactate dehydrogenase" OR LDH OR "physical performance" OR "athletic performance" OR "training adaptation" OR "training adaptations") OR AB=(recover* OR "exercise recovery" OR "recovery intervention" OR "muscle damage" OR "exercise-induced muscle damage" OR EIMD OR DOMS OR "delayed onset muscle soreness" OR soreness OR fatigue OR "oxidative stress" OR inflamm* OR cytokine* OR "C-reactive protein" OR CRP OR "creatine kinase" OR CK OR "lactate dehydrogenase" OR LDH OR "physical performance" OR "athletic performance" OR "training adaptation" OR "training adaptations"))) | 794 |
| SPORTDiscus | 12 June 2026 | (TI (curcumin* OR curcuminoid* OR turmeric OR "Curcuma longa") OR AB (curcumin* OR curcuminoid* OR turmeric OR "Curcuma longa")) AND (TI (exercis* OR sport* OR athlete* OR "physical activity" OR "physical activities" OR "resistance training" OR "strength training" OR "endurance training" OR "aerobic exercise" OR "high-intensity exercise" OR "high intensity interval training" OR HIIT OR running OR runner* OR marathon OR cycling OR soccer OR football OR futsal OR competition OR match) OR AB (exercis* OR sport* OR athlete* OR "physical activity" OR "physical activities" OR "resistance training" OR "strength training" OR "endurance training" OR "aerobic exercise" OR "high-intensity exercise" OR "high intensity interval training" OR HIIT OR running OR runner* OR marathon OR cycling OR soccer OR football OR futsal OR competition OR match)) AND (TI (recover* OR "exercise recovery" OR "recovery intervention" OR "muscle damage" OR "exercise-induced muscle damage" OR EIMD OR DOMS OR "delayed onset muscle soreness" OR soreness OR fatigue OR "oxidative stress" OR inflamm* OR cytokine* OR "C-reactive protein" OR CRP OR "creatine kinase" OR CK OR "lactate dehydrogenase" OR LDH OR "physical performance" OR "athletic performance" OR "training adaptation" OR "training adaptations") OR AB (recover* OR "exercise recovery" OR "recovery intervention" OR "muscle damage" OR "exercise-induced muscle damage" OR EIMD OR DOMS OR "delayed onset muscle soreness" OR soreness OR fatigue OR "oxidative stress" OR inflamm* OR cytokine* OR "C-reactive protein" OR CRP OR "creatine kinase" OR CK OR "lactate dehydrogenase" OR LDH OR "physical performance" OR "athletic performance" OR "training adaptation" OR "training adaptations")) | 66 |
| Cochrane Library/CENTRAL | 12 June 2026 | (curcumin* OR curcuminoid* OR turmeric OR "Curcuma longa"):ti,ab,kw AND (exercis* OR sport* OR athlete* OR "physical activity" OR "physical activities" OR "resistance training" OR "strength training" OR "endurance training" OR "aerobic exercise" OR "high-intensity exercise" OR "high intensity interval training" OR HIIT OR running OR runner* OR marathon OR cycling OR soccer OR football OR futsal OR competition OR match):ti,ab,kw AND (recover* OR "exercise recovery" OR "recovery intervention" OR "muscle damage" OR "exercise-induced muscle damage" OR EIMD OR DOMS OR "delayed onset muscle soreness" OR soreness OR fatigue OR "oxidative stress" OR inflamm* OR cytokine* OR "C-reactive protein" OR CRP OR "creatine kinase" OR CK OR "lactate dehydrogenase" OR LDH OR "physical performance" OR "athletic performance" OR "training adaptation" OR "training adaptations"):ti,ab,kw | 188 |
