# Supplementary material for: Effects of Curcumin Supplementation on Exercise Recovery, Oxidative Stress, Inflammation, Muscle Damage, and Performance in Exercise and Sport Contexts: A Systematic Review
Source: Nutrients. 2026 Jun 19;18(12):1992. doi: 10.3390/nu18121992 (PMC13304679; doi:10.3390/nu18121992)
Supplement: Supplementary file 1 [file nutrients-18-01992-s001.zip › Figure S3.pdf]

Study	D1	DS	D2	D3	D4	D5	Overall
Tanabe et al., 2024	+	-	+	-	+	!	-

+ Low risk

! Some concerns

- High risk

D1 Randomisation process

DS Bias arising from period and carryover effects

D2 Deviations from the intended interventions

D3 Missing outcome data

D4 Measurement of the outcome

D5 Selection of the reported result
