# Supplementary material for: Effects of Curcumin Supplementation on Exercise Recovery, Oxidative Stress, Inflammation, Muscle Damage, and Performance in Exercise and Sport Contexts: A Systematic Review
Source: Nutrients. 2026 Jun 19;18(12):1992. doi: 10.3390/nu18121992 (PMC13304679; doi:10.3390/nu18121992)
Supplement: Supplementary file 1 [file nutrients-18-01992-s001.zip › Figure S4.pdf]

Study	D1	D2	D3	D4	D5	Overall
Bankowski et al., 2025	!	+	-	+	!	-
Faria et al., 2020	+	+	!	+	-	-
Li et al., 2025	+	+	+	+	-	-
Mallard et al., 2021	+	+	+	+	-	-
Salehi et al., 2021	!	+	+	+	!	!
Roohi et al., 2013	!	+	+	+	!	!

+ Low risk

! Some concerns

- High risk

D1 Randomisation process

D2 Deviations from the intended interventions

D3 Missing outcome data

D4 Measurement of the outcome

D5 Selection of the reported result
